# The Impact of a Composite Cardiometabolic Burden on Body Contouring Outcomes: Is the Whole Greater than the Sum of Its Parts?

**DOI:** 10.3390/jcm15114094

**Published:** 2026-05-25

**Authors:** Ron Skorochod, Nir Zontag, Yoram Wolf

**Affiliations:** 1 Unit of Plastic, Reconstructive and Aesthetic Surgery, Hillel Yaffe Medical Center, Hadera 3820302, Israel; nir.zontag@gmail.com (N.Z.); yoramw@hymc.gov.il (Y.W.); 2 Faculty of Medicine, Hebrew University of Jerusalem, Jerusalem 9112102, Israel; 3 Rappaport Faculty of Medicine, Technion—Israel Institute of Technology, Haifa 3200003, Israel

**Keywords:** cardiometabolic burden, body contouring, adverse events, hypertension, obesity, diabetes mellitus

## Abstract

**Background:** Body contouring surgery is a critical aspect of reconstructive and esthetic care, addressing both functional and psychosocial needs. As the global prevalence of obesity and related metabolic disorders is constantly on the rise, it is inevitable that patients presenting for body contouring procedures would display comorbid cardiometabolic conditions that can negatively impact surgical outcomes. Clustered cardiometabolic abnormalities have been linked to increased rates of surgical complications, medical adverse events, prolonged hospitalization, and need for revision procedures. However, its impact on body contouring surgery outcomes remains insufficiently characterized. **Materials and Methods:** TriNetX Global Collaborative Network, comprising deidentified electronic medical records from over 170 healthcare organizations was utilized for this study. Adults undergoing body contouring surgery were stratified by the presence of a composite cardiometabolic burden, defined as the combination of obesity, diabetes mellitus and hypertension, in the year preceding surgery. Cohorts were matched 1:1 using propensity score matching based on baseline demographics, comorbidities, and substance use. Risk ratios with 95% confidence intervals were calculated, with statistical significance set at *p* < 0.05. Outcomes were assessed at 30, 60, and 90 days postoperatively. **Results:** Among 188,164 body contouring patients, 6892 with composite cardiometabolic burden were propensity score–matched to controls. The study group was associated with significantly higher wound complications, surgical site infections, antibiotic use, and emergency department visits at 30, 60, and 90 days postoperatively, with no difference in hypertrophic scarring. **Conclusions**: Composite cardiometabolic burden, as defined in the study, demonstrated a significantly increased risk of adverse events following body contouring surgery, including wound-related morbidity, surgical site infection and increased healthcare utilization. These risks are evident from the early postoperative period and persist through at least the first 90 days after the procedure.

## 1. Introduction

Body contouring surgery is a critical aspect of reconstructive and esthetic care, addressing both functional and psychosocial complaints associated with obesity, massive weight loss, pregnancy or body image dissatisfaction [1,2]. As the global prevalence of obesity and related metabolic disorders is constantly on the rise, it is inevitable that patients presenting for body contouring procedures would display comorbid cardiometabolic conditions that can negatively impact surgical outcomes.

Clustered cardiometabolic abnormalities including obesity, hypertension, impaired glucose metabolism, and dyslipidemia have been associated with impaired wound healing, endothelial stress, increased platelet activity, and chronic low-grade systemic inflammation, mechanisms that are particularly concerning in surgical patients [3,4,5,6].

In other surgical disciplines, clustered cardiometabolic comorbidity has been linked to increased rates of surgical complications, medical adverse events, prolonged hospitalization, and need for revision procedures [7,8,9,10]. However, its impact on body contouring surgery outcomes remains insufficiently characterized.

Previous studies in plastic surgery have namely focused on individual risk factors such as obesity, diabetes, hypertension or smoking when discussing perioperative and postoperative morbidity [11,12,13]. While those variables are important in the analysis of the surgical patient, they fail to provide a complete picture of the synergistic effect of clustered metabolic abnormalities that tend to go hand in hand. This limitation is particularly relevant for body contouring procedures which often involve large tissue manipulations, resection and prolonged operative times, all of which can exacerbate the inherent effect of metabolic irregularities.

Understanding the role of composite cardiometabolic burden as a cumulative risk factor can provide more accurate perioperative risk stratification than evaluation of individual comorbidities, as they are critical for patient selection, preoperative planning, and perioperative care, and especially as demand for body contouring procedures is expected to rise with the constant introduction of weight loss indications for diabetes mellitus agents.

The objective of this study is to evaluate the association between cardiometabolic burden, as a composite variable, and postoperative outcomes following body contouring surgery. By comparing rates of adverse events between the cohorts, we aim to clarify the clinical relevance of metabolic abnormalities in this patient population.

## 2. Methods

### 2.1. Setting

This study is a retrospective cohort study utilizing TriNetX Global Collaborative Network (TriNetX LLC, Cambridge, MA, USA), which contains deidentified electronic medical records from over 170 healthcare organizations.

### 2.2. Ethics

All data accessed through the TriNetX network is anonymized in compliance with the Health Insurance Portability and Accountability Act privacy standards (HIPAA). As the analysis is based solely on previously collected, non-identifiable information, it does not require review by an Institutional Review Board and does not necessitate patient consent under HIPAA provisions.

### 2.3. Cohort Definition

Two cohorts of patients aged 18 and older who underwent body contouring surgery were created. The exposure cohort consisted of patients with concurrent diagnoses of obesity, hypertension, and type 2 diabetes mellitus within the year preceding surgery. Because standardized waist circumference measurements and consistent dyslipidemia coding were not uniformly available across participating healthcare organizations, this operational definition was used to identify patients with clustered cardiometabolic comorbidity rather than formal metabolic syndrome according to consensus diagnostic criteria. The second cohort included patients who underwent the same procedures without the composite exposure diagnosis.

Obesity for cohort construction was identified using BMI-based criteria available within the TriNetX platform.

Body contouring procedures were identified using standardized CPT, ICD-10-PCS, and SNOMED procedural coding and included commonly performed abdominal and truncal contouring procedures, excisional body contouring operations, and related soft tissue contouring interventions. Complete procedural coding definitions are provided in Supplementary Table S1. The index event was defined as the date of surgery.

#### Outcomes

The primary objective was to evaluate whether composite cardiometabolic burden was associated with postoperative outcomes and, second, to determine the duration over which such effects may persist within the early postoperative period. To do so, all outcomes were assessed at three postoperative intervals: within 30 days, 60 days and 90 days.

Wound-related postoperative complications included seroma formation, hematoma, wound dehiscence, wound healing complications, surgical site infection, and hypertrophic scarring. Additional postoperative healthcare utilization and supportive care outcomes included antibiotic prescription, gastrointestinal symptom-related encounters, and emergency department visits. All outcomes were identified using medication and diagnostic coding definitions detailed in Supplementary Table S1.

### 2.4. Statistical Analysis

A 1:1 ratio PSM was conducted using nearest-neighbor matching with a caliper width of 0.1 pooled standard deviations. Matching incorporated baseline characteristics from the day before the index date included demographics, medical conditions, and substance use. Covariate balance following propensity score matching was assessed using standardized mean differences (SMDs), with an SMD < 0.1 considered representative of adequate matching and acceptable balance between cohorts. Table 1 provides the complete list of variables used for matching.

All statistical analyses were performed using the TriNetX platform. For all outcomes, risk ratios (RRs), and 95% confidence intervals (CIs) were calculated. A *p*-value less than 0.05 was considered statistically significant.

## 3. Results

### Study Population and Propensity Score Matching

A total of 188,164 patients undergoing body contouring surgery were identified, including 6892 patients with exposure and 181,272 patients without. Prior to matching, patients in the exposure cohort were significantly older and had higher rates of smoking, and prior bariatric surgery (all *p* < 0.001).

After propensity score matching, all baseline covariates demonstrated adequate balance between cohorts, with standardized mean differences below the predefined threshold of 0.1.

After propensity score matching, 6881 patients remained in each cohort, with adequate covariate balance demonstrated across all matched variables (Table 1).

Within 30 days after surgery, patients with composite cardiometabolic burden demonstrated significantly increased rates of multiple wound-related postoperative complications compared with matched controls (Table 2). These included higher rates of seroma formation (RR 1.47; 95% CI 1.09–1.99), hematoma (RR 1.42; 95% CI 1.14–1.77), wound dehiscence (RR 1.66; 95% CI 1.39–1.98), wound healing complications (RR 1.77; 95% CI 1.33–2.35), and surgical site infection (RR 1.97; 95% CI 1.57–2.47). No statistically significant difference in hypertrophic scarring was identified between cohorts (RR 1.08; 95% CI 0.49–2.37).

Postoperative healthcare utilization and supportive care outcomes were also more frequent among patients with composite cardiometabolic burden. Specifically, exposed patients demonstrated increased rates of postoperative antibiotic prescriptions (RR 1.71; 95% CI 1.54–1.90), gastrointestinal symptom-related encounters (RR 1.93; 95% CI 1.57–2.36), and emergency department visits (RR 1.47; 95% CI 1.32–1.64) within the first 30 postoperative days (Table 2).

At 60 days postoperatively, elevated rates of wound-related complications persisted among patients with composite cardiometabolic burden (Table 3). Compared with matched controls, exposed patients continued to demonstrate increased rates of seroma formation (RR 1.71; 95% CI 1.35–2.16), hematoma (RR 1.50; 95% CI 1.24–1.81), wound dehiscence (RR 1.75; 95% CI 1.51–2.02), wound healing complications (RR 1.94; 95% CI 1.54–2.45), and surgical site infection (RR 1.92; 95% CI 1.59–2.33). Hypertrophic scarring remained comparable between cohorts and did not reach statistical significance (RR 1.36; 95% CI 0.79–2.37).

Similarly, postoperative healthcare utilization outcomes remained increased within the exposure cohort. Patients with composite cardiometabolic burden demonstrated higher rates of postoperative antibiotic prescriptions (RR 1.63; 95% CI 1.48–1.79), gastrointestinal symptom-related encounters (RR 1.82; 95% CI 1.55–2.15), and emergency department visits (RR 1.53; 95% CI 1.39–1.67) at 60 days following surgery (Table 3).

By 90 days postoperatively, patients with composite cardiometabolic burden continued to demonstrate persistently elevated rates of wound-related postoperative morbidity compared with matched controls (Table 4). Increased rates of seroma formation (RR 1.71; 95% CI 1.36–2.14), hematoma (RR 1.56; 95% CI 1.29–1.87), wound dehiscence (RR 1.82; 95% CI 1.58–2.09), wound healing complications (RR 2.05; 95% CI 1.65–2.56), and surgical site infection (RR 1.86; 95% CI 1.55–2.23) remained evident throughout the 90-day postoperative period. No statistically significant difference in hypertrophic scarring was identified between cohorts (RR 1.09; 95% CI 0.68–1.77).

Postoperative healthcare utilization outcomes similarly remained elevated among exposed patients at 90 days after surgery. Composite cardiometabolic burden was associated with increased postoperative antibiotic utilization (RR 1.57; 95% CI 1.44–1.71), gastrointestinal symptom-related encounters (RR 1.78; 95% CI 1.55–2.06), and emergency department visits (RR 1.51; 95% CI 1.39–1.64) compared with matched controls (Table 4).

## 4. Discussion

In this large, multi-institutional, propensity score-matched cohort study, we found composite cardiometabolic burden to be associated with a statistically significant increased risk of postoperative adverse events following body contouring surgery, persisting until 90 days postoperatively. Patients experienced surgical site infection, wound dehiscence, wound healing problems, seroma, hematoma and healthcare services utilization at a greater rate compared to the control group. The consistency of the results over numerous time intervals suggest that it confers a sustained susceptibility to postoperative morbidity rather than a transient perioperative risk.

Importantly, not all evaluated outcomes represented direct surgical complications. Antibiotic prescriptions, gastrointestinal symptom-related encounters, and emergency department visits were interpreted as broader markers of postoperative healthcare utilization and supportive care requirements rather than isolated surgical morbidity.

The relationship between cardiometabolic burden and adverse surgical outcomes has been extensively reviewed in other surgical specialties. It has been shown to impact the rate of postoperative complications, surgical revisions, implant survivorship and length of stay in orthopedic procedures [7,8,14,15,16,17,18], with similar results shown for urological procedures [19,20], general surgery [21,22,23,24,25,26] and neurosurgery [27,28,29].

The association is biologically plausible and most likely driven by multiple synergistic mechanisms. At its core, cardiometabolic burden reflects a state of chronic mild inflammation that leads to insulin resistance, endothelial dysfunction and impaired vascular perfusion, all of which negatively impact wound healing. Insulin resistance and chronic hyperglycemia impair fibroblast proliferation and collagen deposition [30,31], while persistent inflammation inhibits the transition to tissue remodeling state [32]. These pathophysiologic alterations provide a cell-level hypothesis for the consistently elevated rates of wound healing issues and dehiscence found in our cohort.

Emerging evidence additionally suggests that microbiota-associated systemic inflammation and metabolic dysregulation may further contribute to impaired tissue repair and postoperative recovery in metabolically vulnerable patients [33].

The increased risk in surgical site infections observed in patients suffering from cardiometabolic burden further highlights the role of immune dysregulation in this population. Insulin resistance impairs neutrophil chemotaxis and host immune defense while also contributing to endothelial dysfunction and impaired tissue perfusion [34]. Additionally, dyslipidemia fails to inhibit the proinflammatory effect of lipopolysaccharide of Gram-negative bacteria and lipid teichoic acid outer membrane lipid composition of Gram-positive bacteria [35,36].

The persistently higher rate of postoperative antibiotic prescription likely reflects both an increased burden of infectious complications and heightened clinical concern in managing wound-related outcomes in metabolically vulnerable patients. While antibiotics are an important aspect of infection prevention and treatment, these findings underscore the importance of antibiotic stewardship and their appropriate prescription.

Beyond wound-specific outcomes, in our cohort, cardiometabolic burden demonstrated a clear association with increased emergency department visits at all postoperative timepoints. This finding highlights the healthcare burden imposed by cardiometabolic imbalance and suggests that postoperative recovery in this population may be more complex and resource demanding. The increased rate of emergency department visits may reflect an overall higher complication rate, greater symptom burden or increased clinical concern regarding postoperative symptoms.

Interestingly, despite the elevated risk of wound-related complication, it did not demonstrate an association with an increase in hypertrophic scarring. Therefore, it is possible to assume that while metabolic imbalance adversely affects early wound pathology, it may not manifest on later fibroproliferative scar formation. It can also be possible that the follow-up period may not have been sufficient to capture clinically meaningful scar pathology. This distinction highlights the importance of separating early wound healing complications from long-term esthetic outcomes when counseling patients and evaluating surgical success.

From a clinical aspect, these findings have direct implications for the care and management of body contouring surgery patients. Cardiometabolic burden should be recognized as a distinct and clinically relevant risk factor, rather than a collection of isolated comorbidities. Traditional preoperative assessments often focus on individual variables rather than observing the cumulative influence. Our data suggests that the cumulative metabolic burden captured by the composite exposure more accurately identifies patients at increased risk for negative postoperative outcomes. Incorporating cardiometabolic burden into routine preoperative evaluation may improve patient counseling, expectation management, and shared decision-making.

These results also support the implementation of targeted preoperative metabolic optimization for patients diagnosed with metabolic comorbidities. Multidisciplinary approaches aimed at improved glycemic control, hypertension management, nutritional status, and overall optimization of modifiable lifestyle may mitigate the evident postoperative risk. When clinically appropriate, delaying elective body contouring procedures to allow for metabolic optimization may be beneficial. Intraoperatively, physicians may consider tailoring surgical plans by limiting tissue dissection or staging procedures.

Postoperatively, the persistent elevation in healthcare utilization highlights the importance of enhanced surveillance and follow-up in patients with greater surgical risk. Closer monitoring, earlier and more frequent wound assessments and lower thresholds for intervention may prevent minor wound issues from progressing into more severe complications and need for unplanned reoperation or emergency care.

Several limitations of this study must be acknowledged. First, this was a retrospective observational study utilizing deidentified electronic healthcare record data from a large, federated database and is therefore inherently limited by data granularity and potential coding inaccuracies. Consequently, the possibility of misclassification bias related to diagnostic and procedural coding remains. Second, although propensity score matching was performed to improve baseline comparability between cohorts, residual confounding related to unmeasured variables cannot be fully excluded. Granular perioperative and metabolic variables including continuous BMI measurements, hemoglobin A1c levels, nutritional status, operative duration, extent of tissue dissection, drain utilization, and surgeon-specific or institution-specific practices were not consistently available within the database and therefore could not be incorporated into the analysis.

Third, the exposure definition used in this study does not fully replicate established consensus diagnostic criteria for metabolic syndrome, which classically incorporate additional components such as dyslipidemia and waist circumference measurements. Due to limitations in the consistent availability and standardization of these variables across participating healthcare organizations, the exposure cohort should be interpreted as representing patients with clustered cardiometabolic comorbidity rather than formally diagnosed metabolic syndrome according to consensus definitions. Fourth, body contouring surgery encompasses a heterogeneous group of procedures with varying operative complexity, extent of tissue manipulation, and baseline complication profiles. Although subgroup analyses according to individual procedure subtype may provide additional clinical granularity, stratification of the matched cohorts resulted in insufficient case numbers and low event frequencies for reliable outcome assessment across multiple postoperative endpoints and follow-up intervals. Finally, patient-reported outcomes and long-term esthetic satisfaction measures were not available within the TriNetX platform and therefore could not be assessed.

Despite these limitations, our study leverages a large, multi-institutional, well-matched cohort for longitudinal assessment of outcomes and adverse events. Recognition of composite cardiometabolic burden as a clinically relevant and potentially modifiable risk profile provides a unique opportunity to improve patient outcomes and adequately estimate the potential for adverse events in a critical population of patients.

Importantly, the associations identified in this study were not only statistically significant, but also clinically meaningful. Patients with clustered cardiometabolic comorbidity demonstrated nearly two-fold higher risks of surgical site infection and wound healing complications, in addition to persistently elevated rates of wound dehiscence, seroma, hematoma, postoperative antibiotic utilization, and emergency department visits across all evaluated postoperative intervals. In body contouring surgery, where postoperative wound integrity and recovery are central determinants of both reconstructive and esthetic success, these magnitudes of risk likely represent substantial clinical burden rather than isolated statistical associations. Furthermore, the consistency of elevated risk across multiple independent complication domains and throughout all postoperative timepoints strengthens the clinical relevance of these findings. Rather than reflecting transient perioperative events alone, the results suggest a sustained postoperative vulnerability among patients with increased cardiometabolic burden.

These findings may have important implications for perioperative counseling, patient selection, postoperative surveillance, and consideration of targeted preoperative metabolic optimization strategies in higher-risk body contouring candidates.

In conclusion, cardiometabolic burden is associated with a significantly increased risk of adverse events following body contouring surgery, including wound-related morbidity, surgical site infection and increased healthcare utilization. These risks are evident from the early postoperative period and persist through at least the first 90 days after the procedure.

Recognition of cardiometabolic burden as a distinct, cumulative risk profile may enhance preoperative risk stratification, patient counseling and postoperative outcomes. Targeted metabolic optimization in the preoperative setting, enhanced postoperative surveillance and early intervention in occurring complications represent potential strategies to mitigate risk and improve outcomes in this high-risk patient population.

## Figures and Tables

**Table 1:** Baseline variables used for propensity score matching and between-group differences.

	Before Matching			After Matching		
Variable	Composite Cardiometabolic Burden (n = 6892)	No Composite Cardiometabolic Burden (n = 181,272)	SMD	Composite Cardiometabolic Burden (n = 6881)	Composite Cardiometabolic Burden (n = 6881)	SMD
Age	57.1 + 11.2	44.8 + 17.1	0.86	57.2 + 11.2	57.3 + 11.4	0.008
Male	1362	25,505	0.15	1360	1298	0.02
Female	5520	152,777	0.14	5520	5579	0.02
White	4520	119,982	0.03	4520	4595	0.02
African American	1756	28,322	0.24	1754	1744	0.03
Hispanic	920	17,679	0.11	920	909	0.01
Asian	61	4580	0.13	61	53	0.013
American Indian or Alaskan	36	713	0.02	36	32	0.002
Native Hawaiian	33	711	0.01	33	25	0.01
Unknown Ethnicity	1194	38,357	0.10	1194	1185	0.04
Unknown Race	291	16,977	0.21	291	264	0.02
Smoking	1845	10,331	0.45	1844	1859	0.005
Prior Bariatric Surgery	1800	16,301	0.59	1798	1747	0.02

**Table 2:** Adverse events and outcomes 30 days after surgery.

	Outcome	Composite Cardiometabolic Burden	No Composite Cardiometabolic Burden	RR	95% CI
Wound-Related outcomes	Seroma	103	70	1.47	1.09–1.99
	Hematoma	186	131	1.42	1.14–1.77
	Wound Dehiscence	319	192	1.66	1.39–1.98
	Wound Healing Problem	131	74	1.77	1.33–2.35
	Surgical Site Infection	217	110	1.97	1.57–2.47
	Hypertrophic Scarring	13	12	1.08	0.49–2.37
Healthcare Utilization	Antibiotic Prescription	861	504	1.71	1.54–1.90
	GI Symptoms	260	135	1.93	1.57–2.36
	Emergency Department Visit	759	515	1.47	1.32–1.64

**Table 3:** Adverse events and outcomes 60 days after surgery.

	Outcome	Composite Cardiometabolic Burden	No Composite Cardiometabolic Burden	RR	95% CI
Wound-Related outcomes	Seroma	181	106	1.71	1.35–2.16
	Hematoma	258	172	1.5	1.24–1.81
	Wound Dehiscence	474	271	1.75	1.51–2.02
	Wound Healing Problem	206	106	1.94	1.54–2.45
	Surgical Site Infection	296	154	1.92	1.59–2.33
	Hypertrophic Scarring	30	22	1.36	0.79–2.37
Healthcare Utilization	Antibiotic Prescription	1030	633	1.63	1.48–1.79
	GI Symptoms	387	212	1.82	1.55–2.15
	Emergency Department Visit	1036	678	1.53	1.39–1.67

**Table 4:** Adverse events and outcomes 90 days after surgery.

	Outcome	Composite Cardiometabolic Burden	No Composite Cardiometabolic Burden	RR	95% CI
Wound-Related outcomes	Seroma	200	117	1.71	1.36–2.14
	Hematoma	280	180	1.56	1.29–1.87
	Wound Dehiscence	529	291	1.82	1.58–2.09
	Wound Healing Problem	234	114	2.05	1.65–2.56
	Surgical Site Infection	323	174	1.86	1.55–2.23
	Hypertrophic Scarring	35	32	1.09	0.68–1.77
Healthcare Utilization	Antibiotic Prescription	1176	751	1.57	1.44–1.71
	GI Symptoms	499	280	1.78	1.55–2.06
	Emergency Department Visit	1195	794	1.51	1.39–1.64

## Data Availability

The original contributions presented in this study are included in the article/Supplementary Materials. Further inquiries can be directed to the corresponding author.

## Supplementary Materials

The following supporting information can be downloaded at: https://www.mdpi.com/article/10.3390/jcm15114094/s1, Table S1: The complete list of codes for inclusion and exclusion criteria used for cohort composition.
