# Supplementary material for: The Impact of a Composite Cardiometabolic Burden on Body Contouring Outcomes: Is the Whole Greater than the Sum of Its Parts?
Source: J Clin Med. 2026 May 25;15(11):4094. doi: 10.3390/jcm15114094 (PMC13258387; doi:10.3390/jcm15114094)
Supplement: Supplementary file 1 [file jcm-15-04094-s001.zip › jcm-4321297-supplementary.pdf]

Supplementary Table S1: The complete list of codes for inclusion and exclusion criteria used for cohort composition.

	CODE	DEFENITION
**ICD-10**	E65-E68	Overweight, obesity and other hyperalimentation
**ICD-10**	E11	Type 2 Diabetes Mellitus
**ICD-10**	I10	Essential (Primary) Hypertension
**CPT**	19316	mastopexy
**CPT**	19300	Mastectomy for gynecomastia
**CPT**	15837	Excision, excessive skin and subcutaneous tissue (includes lipectomy); forearm or hand
**CPT**	15833	Excision, excessive skin and subcutaneous tissue (includes lipectomy); leg
**CPT**	15838	Excision, excessive skin and subcutaneous tissue (includes lipectomy); submental fat pad
**CPT**	15835	Excision, excessive skin and subcutaneous tissue (includes lipectomy); buttock
**CPT**	15834	Excision, excessive skin and subcutaneous tissue (includes lipectomy); hip
**CPT**	15836	Excision, excessive skin and subcutaneous tissue (includes lipectomy); arm
**CPT**	15839	Excision, excessive skin and subcutaneous tissue (includes lipectomy); other area
**CPT**	15832	Excision, excessive skin and subcutaneous tissue (includes lipectomy); thigh
**CPT**	15847	Excision, excessive skin and subcutaneous tissue (includes lipectomy), abdomen (eg, abdominoplasty) (includes umbilical transposition and fascial plication) (List separately in addition to code for primary procedure)
**CPT**	15830	Excision, excessive skin and subcutaneous tissue (includes lipectomy); abdomen, infraumbilical panniculectomy
**CPT**	193018	Breast Reduction
